# Supplementary material for: IL‐10 producing type 2 innate lymphoid cells prolong islet allograft survival
Source: EMBO Mol Med. 2020 Oct 9;12(11):e12305. doi: 10.15252/emmm.202012305 (PMC7645373; doi:10.15252/emmm.202012305)
Supplement: Supplementary file 1 — Appendix [file EMMM-12-e12305-s001.pdf]

Appendix

Content:

Appendix Fig. S1: IL-33 treatment improved glycemia, but not survival rate in STZ-induced diabetic mice.

Appendix Fig. S2: IL-33 induce a Th1-to-Th2 switch in vivo.

Appendix Fig. S3: Distribution of ILC2s in mice treated with IL-33.

Appendix Fig. S4: IL-33 enhanced the expression of CD25 in ILC2s.

Appendix Fig. S5: The expression of signature cytokines in locally transplanted $ILC2^{10}$.

Appendix Table S1: PCR primer sequences.

Appendix Table S2: List of exact P-values.

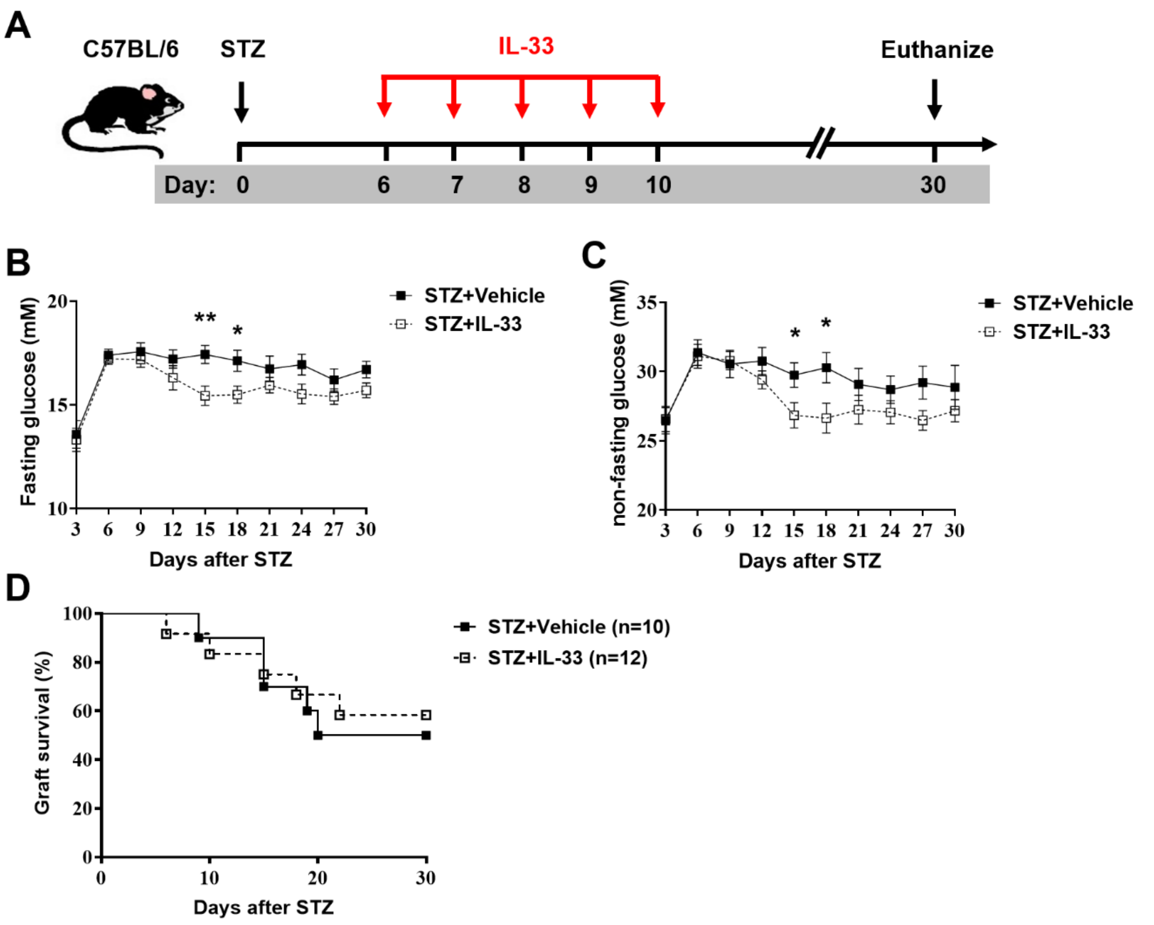

Appendix Figure S1. IL-33 treatment improved glycemia, but not survival rate in STZ-induced diabetic mice.

(A) STZ-induced diabetic C57BL/6 mice were treated with vehicle or IL-33 daily from day 6 to 10 post-STZ injection. (B and C) Fasting and non-fasting blood glucose concentrations were monitored. Data shown are the mean ± SEM (n=7-9 per group) and an unpaired t-test was performed. *P<0.05, **P<0.01. (D) Survival rate of diabetic C57BL/6 mice treated with vehicle or IL-33.

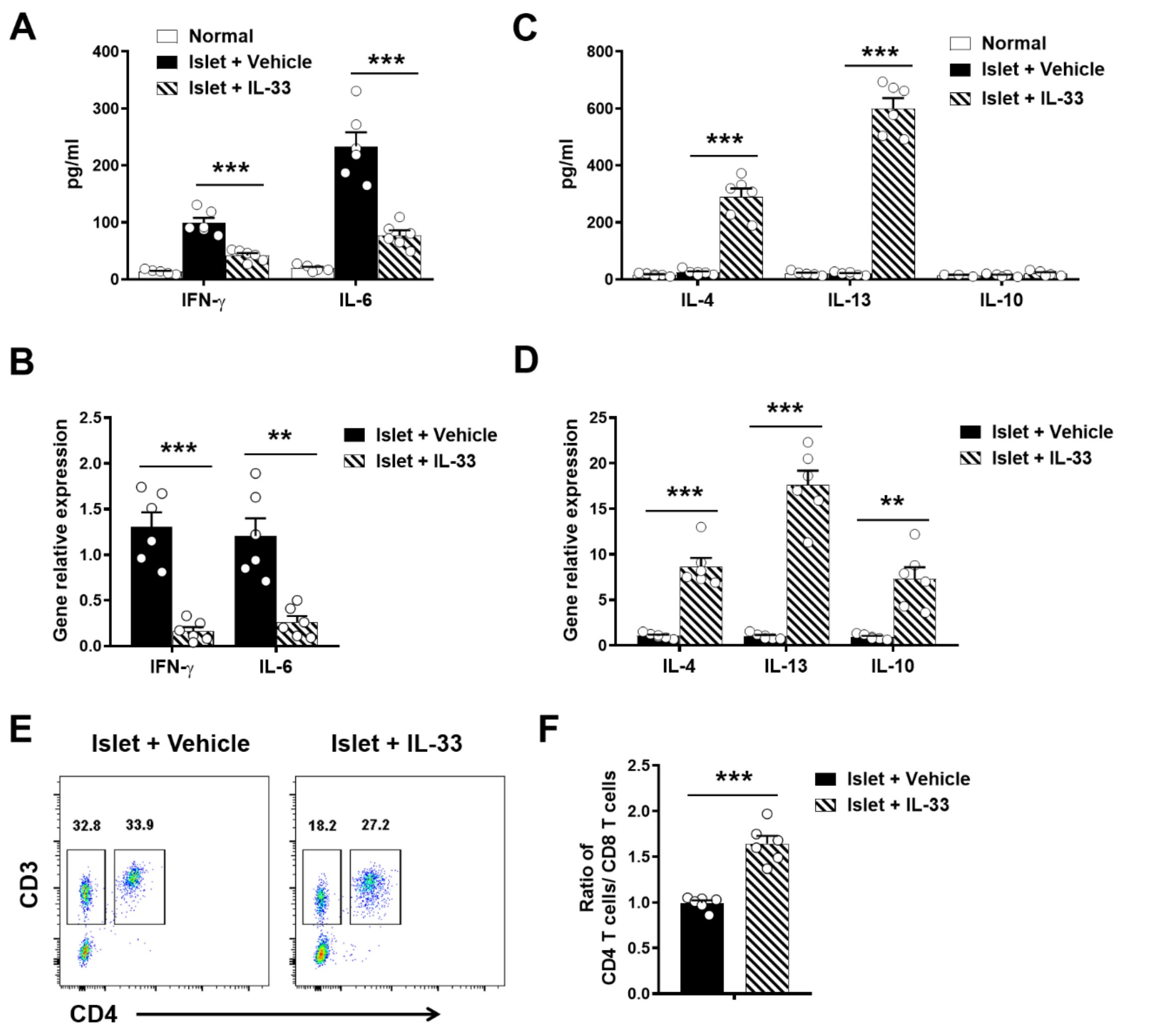

Appendix Figure S2. IL-33 induce a Th1-to-Th2 switch in vivo.

(A and C) IFN-γ, IL-6, IL-4, IL-10 and IL-13 levels in serum were assessed in normal, Islet+Vehicle and Islet+IL-33 groups at day 5 post islet transplantation. (B and D) The mRNA expression of IFN-γ, IL-6, IL-4, IL-10 and IL-13 in islet allografts was examined by qPCR, and expressed relative to the control of each experiment. Data shown are the mean ± SEM (n=6 per group) and an unpaired t-test was performed. **P<0.01, ***P<0.001. (E) Representative FACS analysis showing the proportion of CD4+ T cells, and CD8+ T cells (CD3+CD4- cells) in the CD45+ leukocyte compartment of islet graft of mice receiving vehicle or IL-33. (F) Ratio of CD4 T cells/CD8 T cells in islet graft of mice receiving vehicle or IL-33. ***P<0.001.

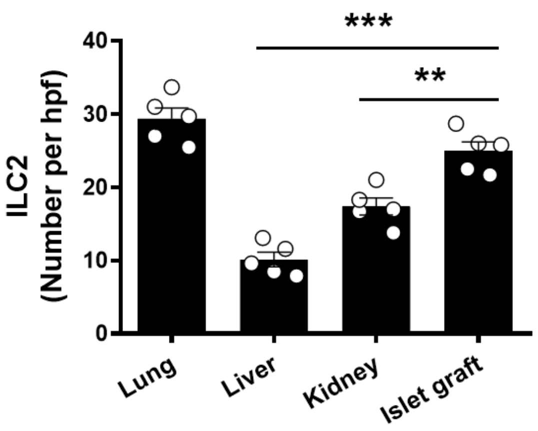

Appendix Figure S3. Distribution of ILC2s in mice treated with IL-33.

Streptozotocin-induced diabetic C57BL/6 mice with IL-33 treatment were transplanted with BALB/c islets. Mice were sacrificed at day 5 post-islet transplantation. The numbers of CD3(−)CD127+ST2+ ILC2s in lung, liver, kidney and islet graft were counted. Data shown are the mean ± SEM (n=5 per group) and a one-way ANOVA was performed; **P<0.01, ***P<0.001.

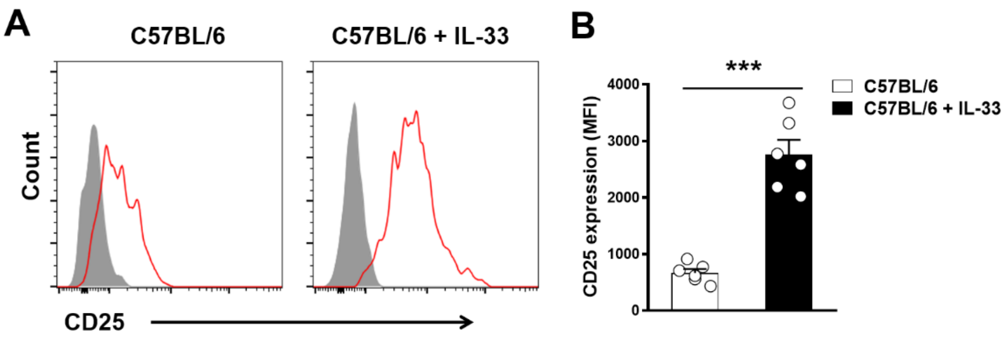

Appendix Figure S4. IL-33 enhanced the expression of CD25 in ILC2s.

C57BL/6 mice were treated with mouse recombinant IL-33 or PBS daily for 5 consecutive days. (A and B) The expression of CD25 was examined in kidney ILC2s by flow cytometry. ILC2s (red lines) and isotype controls (gray-filled areas) are shown. Data represent the mean ± SEM of evaluations of MFI from each group (n=6 per group) and an unpaired t-test was performed. ***P<0.001. MFI, mean fluorescence intensity.

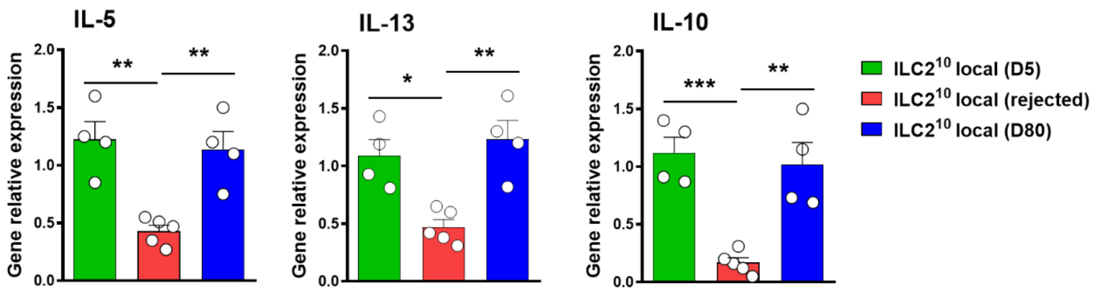

Appendix Figure S5. The expression of signature cytokines in locally transplanted ILC2^{10}.

The locally transplanted CD45.2+ST2+ ILC2^{10} were isolated from islet grafts by flow sorting at day 5 and 80 post-islet transplantation or at the day when grafts were considered rejected. The mRNA expression of IL-5, IL-13 and IL-10 in locally transplanted ILC2^{10} was examined by qPCR, and expressed relative to the control of each experiment. Data shown are the mean ± SEM (n=4-5 per group) and a one-way ANOVA was performed. **P<0.01, ***P<0.001.

Appendix Table S1. Real-time PCR primers

Gene	Forward (5’-3’)	Reverse (5’-3’)
IL-4	tcaacccccagctagttgtc	tctgtggtgttcttcgttgc
IL-5	aaagagaagtgtggcgaggag	tcaccatggagcagctcag
IL-13	cagcatggtatggagtgtgg	aggctggagaccgtagtgg
IL10	ccagtacagccgggaagaca	cagctggtcctttgtttgaaaga
IFN-γ	gcgtcattgaatcacacctg	acctgtgggttgttgacctc
IL-6	cacaagtccggagaggagac	ttgccattgcacaactcttt
IL-25	tggagctatgagttggacagg	gaagaccgtctggttgtggt
IL-33	acattgagcatccaaggaactt	gcgtagtagcacctggtcttg
TSLP	aggctaccctgaaactgag	ggagattgcatgaaggaatacc

TSLP: thymic stromal lymphopoietin

Appendix Table S2. List of exact P-values

Figure	Panel	sub-panel	compared groups, P-value
Figure 1	B		Islet+IL-33 vs Islet+vehicle <0.0001
	E		Islet+IL-33 (D30) vs Islet+vehicle <0.0001<br>Islet+IL-33 (D30) vs Islet+vehicle <0.0001
Figure 2	A		IL-33 vs PBS <0.0001
	B		IL-33 vs PBS 0.0006
	C		IL-33 vs PBS 0.0030
	D		IL-33 vs PBS <0.0001
Figure 3	B		Islet+IL-33 (D7) vs Islet+vehicle <0.0001
	C		Islet+IL-33 (D7) vs Islet+vehicle <0.0001
	D		Islet+IL-33 (D7) vs Islet+vehicle <0.0001
	F		Islet+IL-33 (D7) vs Islet+vehicle <0.0001
	G		Islet+IL-33 (D7) vs Islet+vehicle <0.0001<br>Islet+IL-33 (D30) vs Islet+vehicle <0.0001<br>Islet+IL-33 (D80) vs Islet+vehicle 0.0055
	H		Islet+IL-33 (D7) vs Islet+vehicle <0.0001<br>Islet+IL-33 (D30) vs Islet+vehicle <0.0001<br>Islet+IL-33 (D80) vs Islet+vehicle <0.0001
	J	IL-33	Islet+IL-33 (D7) vs Islet+vehicle <0.0001<br>Islet+IL-33 (D30) vs Islet+vehicle 0.0004<br>Islet+IL-33 (D80) vs Islet+vehicle 0.0020
Figure 4	B	Spleen	Islet+IL-33/DT vs Islet+IL-33 <0.0001<br>Islet+IL-33/PC61 vs Islet+IL-33 <0.0001<br>Islet+IL-33/DT/PC61 vs Islet+IL-33 <0.0001
		Kidney	Islet+IL-33/DT vs Islet+IL-33 <0.0001<br>Islet+IL-33/PC61 vs Islet+IL-33 <0.0001<br>Islet+IL-33/DT/PC61 vs Islet+IL-33 <0.0001
	C	Spleen	Islet+IL-33/PC61 vs Islet+IL-33 0.0003

			Islet+IL-33/DT/PC61 vs Islet+IL-33 0.0005
		Kidney	Islet+IL-33/PC61 vs Islet+IL-33 <0.0001<br>Islet+IL-33/DT/PC61 vs Islet+IL-33 <0.0001
	D		Islet+IL-33/DT vs Islet+IL-33 0.0280<br>Islet+IL-33/PC61 vs Islet+IL-33/DT 0.0086<br>Islet+IL-33/DT/PC61 vs Islet+IL-33/DT 0.0066
Figure 5	A		Islet+IL-33 vs Islet+vehicle 0.0002
	B		Islet+IL-33 vs Islet+vehicle 0.0005
	C		IL-33/IL-2C vs IL-33 <0.0001
	D		IL-33/IL-2C vs Medium <0.0001
	E		IL-33/IL-2C vs Medium 0.0002
	F		IL-33/IL-2C vs Medium 0.0001
	G		IL-33/IL-2C+STAT5-IN vs IL-33/IL-2C <0.0001
Figure 6	A		ILC2^{10}-IL-10 vs ILC2^{10}-C <0.0001
	D		Islet+ILC2^{10}-C vs Islet+vehicle 0.0005<br>Islet+ILC2^{10}-IL-10 vs Islet+ILC2^{10}-C 0.0007<br>Islet+non-ILC2^{10} vs Islet+ILC2^{10}-C 0.0016
	E		Islet+ILC2^{10}-C vs Islet+vehicle <0.0001<br>Islet+ILC2^{10}-IL-10 vs Islet+ILC2^{10}-C <0.0001<br>Islet+non-ILC2^{10} vs Islet+ILC2^{10}-C <0.0001
	G		CD4+ILC2^{10}+anti-IL-10 vs CD4+ILC2^{10} 0.0003<br>CD4+ILC2^{10}-IL-10 vs CD4+ILC2^{10}-C <0.0001
Figure 7	B		Islet+ILC2^{10} local (1x10^{6}) vs Islet+ ILC2^{10} iv 0.0002<br>Islet+ILC2^{10} local (2x10^{5}) vs Islet+ ILC2^{10} iv 0.0009<br>Islet+ ILC2^{10}-IL-10 local (2x10^{5}) vs Islet+ILC2^{10} local (2x10^{5}) <0.0001
	D		ILC2^{10} local (rejected) vs ILC2^{10} local (D5) <0.0001<br>ILC2^{10} local (D80) vs ILC2^{10} local (D5) 0.0031<br>ILC2^{10} local (D80) vs ILC2^{10} local (rejected) 0.0080
	E		ILC2^{10} local (D80) vs ILC2^{10} local (D5) 0.0019

			ILC2^{10} local (D80) vs ILC2^{10} local (rejected) 0.0015
	F		ILC2^{10} local (rejected) vs ILC2^{10} local (D5) <0.0001<br>ILC2^{10} local (D80) vs ILC2^{10} local (rejected) <0.0001
Appendix Fig. S1	B	D15	STZ+IL-33 vs STZ+vehicle 0.0075
		D18	STZ+IL-33 vs STZ+vehicle 0.0260
	C	D15	STZ+IL-33 vs STZ+vehicle 0.0400
		D18	STZ+IL-33 vs STZ+vehicle 0.0335
Appendix Fig. S2	A	IFN-γ	Islet+IL-33 vs Islet+vehicle 0.0004
		IL-6	Islet+IL-33 vs Islet+vehicle 0.0008
	B	IFN-γ	Islet+IL-33 vs Islet+vehicle 0.0005
		IL-6	Islet+IL-33 vs Islet+vehicle 0.0031
	C	IL-4	Islet+IL-33 vs Islet+vehicle 0.0002
		IL-13	Islet+IL-33 vs Islet+vehicle <0.0001
	D	IL-4	Islet+IL-33 vs Islet+vehicle 0.0004
		IL-13	Islet+IL-33 vs Islet+vehicle 0.0001
		IL-10	Islet+IL-33 vs Islet+vehicle 0.0042
	F		Islet+IL-33 vs Islet+vehicle 0.0003
Appendix Fig. S3			Kidney vs Liver 0.0025<br>Islet graft vs Liver <0.0001
Appendix Fig. S4	B		C57BL/6+IL-33 vs C57BL/6 <0.0001
Appendix Fig. S5		IL-5	ILC2^{10} local (rejected) vs ILC2^{10} local (D5) 0.0021<br>ILC2^{10} local (D80) vs ILC2^{10} local (rejected) 0.0046
		IL-13	ILC2^{10} local (rejected) vs ILC2^{10} local (D5) 0.0111<br>ILC2^{10} local (D80) vs ILC2^{10} local (rejected) 0.0030
		IL-10	ILC2^{10} local (rejected) vs ILC2^{10} local (D5) 0.0008<br>ILC2^{10} local (D80) vs ILC2^{10} local (rejected) 0.0019
